# The role of direct contact in forming peace-oriented international relations of a divided country: Focusing on the division of the Korean Peninsula

**DOI:** 10.1371/journal.pone.0287666

**Published:** 2023-11-22

**Authors:** Hye Cho Shin, Gi-Eun Lee, Seo-Yun Choi, Jang-Han Lee

**Affiliations:** 1 Foreign Studies Institute, Chung-Ang University, Seoul, Republic of Korea; 2 Department of Psychology, Chung-Ang University, Seoul, Republic of Korea; Universidade Federal de Minas Gerais, BRAZIL

## Abstract

**Background:**

The division of the Korean peninsula involved many neighbouring countries in the Korean War. The relations with those countries have since been reorganised due to active exchange. This study examined how the quantity and quality of contact with traditional alliance (US and Japan) and strategic partner (China and Russia) countries affected their national images.

**Methods:**

To this end, this study analyzed the relation with the national image by measuring the quantity and quality of contact of an individual with each country. The quantity of contact included an evaluation of the individual’s subjective amount of contact, contact path, and contact status, and the quality of contact was measured as an evaluation for the pleasure, competitiveness, intimacy, spontaneity, and necessity when contacting each country’s culture. A total of 387 participants were divided into two groups based on the presence of direct contact and the quantity and quality of their contact and national images were examined. The participants were followed by a completion of the self-questionnaires including the Culture Experience Questionnaire, National Image Questionnaire, and demographic information questionnaire.

**Results:**

The results of this study are as follow: first, regardless of the type of country, the national image was highly correlated with the degree of subjective contact evaluated by individuals, but there was a weak tendency with contact quality. Second, there was no significant interaction between the country type and contact status for national image, however, different national images for each country were detected. In other word, for contact quantity, contact groups showed more positive national images compared to non-contact groups in Russia, but not Japan, China, and the US. For contact quality, the positive contact experience group showed more positive national images compared to the negative contact experience group, but only in traditional alliance countries.

**Conclusion:**

This study highlights the importance of implementing different strategies for countries to maintain peaceful international relations.

## Introduction

On April 27, 2018, the supreme leader of North Korea, Kim Jong-un, stepped into South Korea [1]. It was the first time a North Korean leader had done so since the armistice was signed in 1953. Thousands of press members across the world shot camera flashes as many countries were interested in the divided Korean nation. Neighbouring countries showed a particular interest in this situation because these countries, along with the United States, Japan, China, and Russia, were also involved in the Korean War and had directly affected the division of the Korean peninsula. In particular, the four major countries mentioned above are currently involved in direct international relations with South Korea. Because of the potential denuclearisation of North Korea, these relations are very complex. The relation between South Korea and North Korea could directly affect all four countries.

South Korea has maintained traditional alliances with the US and Japan through economic, political, and cultural exchanges [2]. However, unlike the USA, Korea, which received Japanese colonial era in the past, has a somewhat negative image of Japan. Japan has been at odds with Korea as it continued to debate the issue of territorial marking on maps and the denial of the Japanese Military Sexual Slavery incident of colonial Japanese soldiers [3–5]. Nevertheless, Japan is considered to have a friendly and allied relation with China and Russia in that it has a political system similar to that of Korea. On the other hand, China and Russia have had confrontational relations with Korea because of their alliances with North Korea [2]. Although both countries have been lacking ties with Korea, there is a difference between those relations because of the geographical proximity of China, which has led to many international cultural and human exchanges. In this way, the relation between Korea and these four countries can be largely divided into dichotomy of mutual relations that are ‘confrontational’ (i.e. China and Russia) or ‘friendly’ (i.e. USA and Japan) depending on the historical process [6]. Traditionally, it was considered a simple relation due to this dichotomous attitude and perspective of each country, but the recent development of the Internet or science and technology has increased the opportunity for contact due to the diversification of the world where culture and culture can be easily exchanged. Nonetheless, dualistic images on these countries may still exist because of past relations.

A national image is a set of beliefs and impressions that people hold about a country or its people [7, 8]. It is unclear whether such a national image affects actual behaviour related to a nation. For example, even if a person holds a negative national image of a nation, they can travel to that nation to determine their feelings. However, the national image more significantly recognised through symbolic and emotional factors is expected to affect various aspects of international relations [9]. This national image may be formed by the information or knowledge that an individual already has or may change depending on how the individual contacted each country and its culture.

According to the contact theory, directly experiencing a culture through residence or visitation has a positive effect on the perception of national image [10–18], because meaningful experience with outgroup members can reduce stereotypic images [19]. However, direct contact experiences do not always have positive effects, as experiences are not always meaningful and positive; outcomes may vary depending on the context and time in which the contact occurs or the characteristics of the contact group [10, 11, 20–22]. For example, national images of certain country became more positive after people had actual contact with culture or people of country [23]. Furthermore, the level of amount of contact experiences (e.g., studying abroad or traveling) have resulted in more favourable national images [23, 24]. Especially for the USA, the quality of direct contact experiences (e.g., close relations) more directly impacts attitudes towards the American nation and people than the quantity of experiences (e.g., how often meetings take place) [25].

The Common Ingroup Identity Model [26] also argues that this bias by contact can be reclassified because intergroup bias is based on the classification of internal and external groups. Therefore, it can be estimated that the experience of contact with each country can affect changing the image of each country. The previous research indicating that direct contact (e.g., traveling and studying abroad) positively affected national images of the USA and Japan [23, 24]. However, one of these previous studies asked participants who had studied in Japan to conduct retrospective tests, with a limitation in that they did not have sufficient control over the national image of Japan before studying abroad [24]. In other words, the contact effect on the national image was evaluated in a mixture of memories before and after experiencing Japanese culture. Therefore, the results of this study have a limitation in that it is difficult to say that the national image derived from actual experience was evaluated because knowledge of Japan may have influenced the judgments of the national image before contacting the actual Japanese culture. In addition, there is an individual level of motivation that affects the migration intentions of individuals who want to move from one country to another. In particular, factors such as the social life desired by an individual who wants to migrate may be influential, which would prefer to migrate when there is a proud, close social community, well-educated, or strong ties to his or her country. This suggests that individual level motivation may be affected by migration intentions [27, 28]. However, a study that confirmed the effects of direct contact among people who had travelled to the USA neglected to compare these participants with people who had no contact experience [23]. Moreover, other previous studies have not confirmed whether the effects of direct contact differ depending on the characteristics of each country. However, research has indicated that direct contact may not have the same positive impact on all countries [21]. Korea has formed different types of international relations with the four countries most closely related to Korea: traditional alliances and strategic partnerships. The characteristics of its relations with each type differ because of varying historical, cultural, and social experiences. Therefore, the effects of direct contact should be verified by the same measure of national image perceptions on four countries with different characteristics.

According to the framework of attitude theory, understanding of the national image can be expressed using three representative factors: attitude, belief, influence, and behavior [29, 30]. Emotional elements of attitudes provide an opportunity for images to interact when people participate in cognitive processing of information during the evaluation phase. The emotional elements of the model are represented by the evaluation of destinations, the attractiveness of national contacts, and the state itself. Because the destination dimension is part of the overall image and represents part of the overall lifestyle of people’s lives, the state as a destination is likely to affect the overall evaluation of the country. The attractiveness of country-specific contact is part of the overall national image and dimensions can be well related to each other, and prior studies suggest that it is related to the overall evaluation [31]. The final element of action in this model represents the intention of potential traveler migrants to choose the country to live and work in. The overall national assessment is expected to be related to behavioral intentions driven by attitude theory.

To confirm this, this study examined the effect of the quantity and quality of direct contact between traditional alliance (i.e. the USA and Japan) and strategic partner (i.e. Russia and China) countries on their national images. On the other hand, in previous studies, contact with each country and research on national image were conducted, but there is a limitation that it was not conducted in consideration of strategic or friendly relations between each country. Therefore, this study attempted to explore how the existing biased attitude toward each neighboring country can vary depending on the quantity and quality of contact with each country based on Korean.

Unlike generations of people who directly experienced international relations due to past geopolitical contact, young adults in Korea have not experienced the Korean war or other historical situations. Nonetheless, the national images of other countries have been formed by textbooks, mass media, and stories about international relations involving older generations. However, young Korean adults are increasingly replacing voyeuristic fantasies and prejudices towards other nations by traveling to other countries, exchanging college credits, and participating in internships abroad. Another interesting discovery is that, despite active contact experiences, national images have not changed much about some countries because of previous education and storytelling from older generations. Opportunities for direct contact have thus increased, but it is still necessary to confirm the national images that younger generations of Koreans hold about other countries because negative attitudes may persist because of information passed down by previous generations.

**Fig 1:**
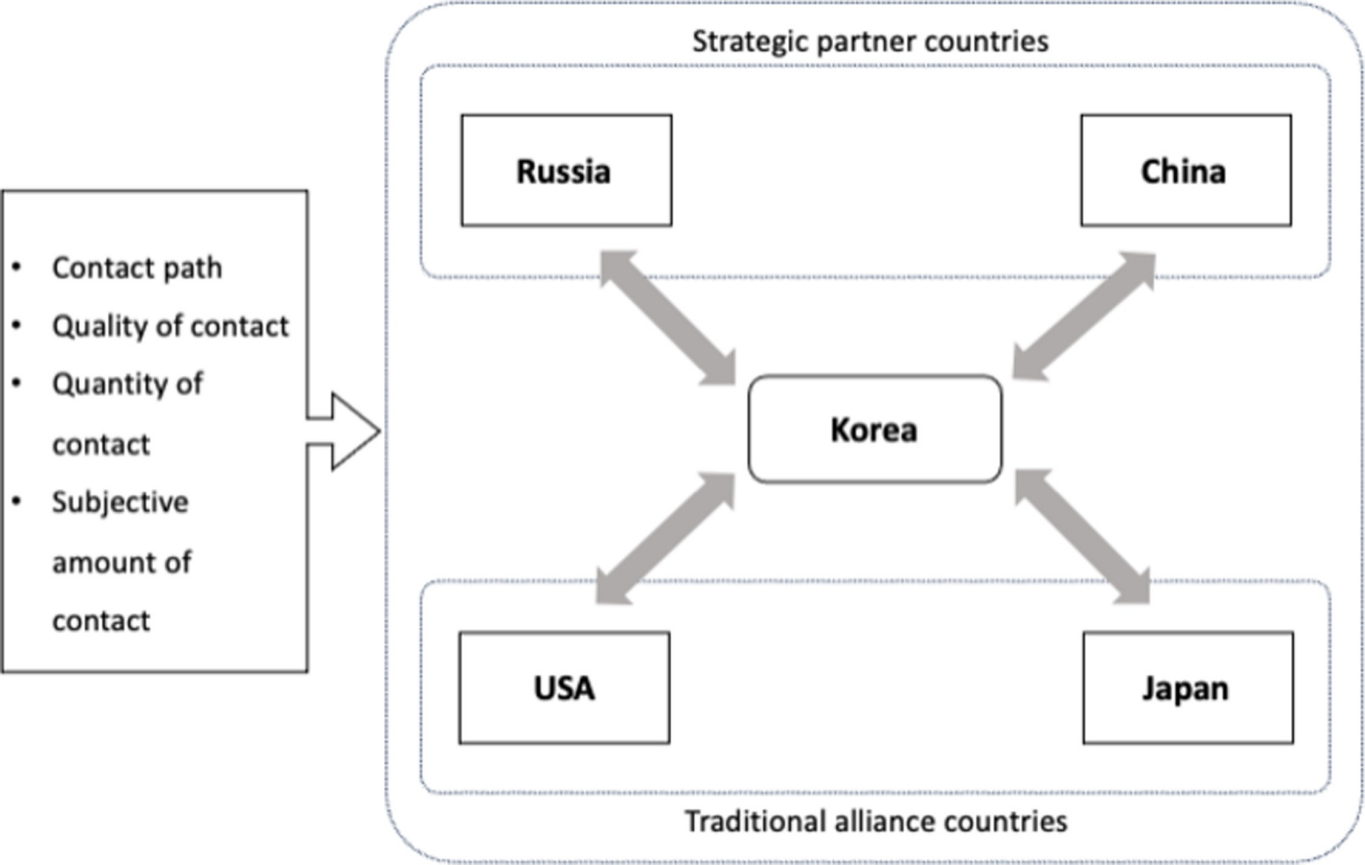
Country image by country relationship and type of contact.

In summary, present study is an exploratory study to examine the effect of the quantity and quality of direct contact between Koreans who experiencing ceasefire in divided countries and people from traditional and strategic partner countries on their national images. To do this, the relations with the USA and Japan is described as traditional alliances, and those with China and Russia is described as strategic partners in this study. Strategic partner countries can positively impact the national image through the quantity of contact. However, this is insufficient for traditional alliance countries, which should focus on the quality of contact when attempting to influence the national image. That is, positive contact can result in positive national images, while negative contact can result in negative national images. It is hypothesized that the national image of strategic partner countries is affected by the quantity of contact, whereas the national image of traditional alliance countries is affected by the quality of contact. Thus, we expect that the more contact there is, the more positive national images of China and Russia, and the more positive contact there is, the more positive national images of Japan and the US (Fig 1).

## Methods

### Participants

The participants were 387 South Korean students aged in their 20s who were recruited from the university’s online bulletin to assess the national image of both traditional alliance and strategic partner countries (i.e. USA, Russia, Japan, and China). Since the degree of cultural experience in other countries may be affected by the level of education, all participants were selected only for college students. Also, only people whose families were not foreigners or whose families did not live abroad could participate in the study. Subjects were divided into “contact” and “non-contact” groups through a pre-screening process asking whether they had experience to contact with each country’s culture. According to previous study [32], those who only passed individuals of each nationality on the street or seen them in restaurants were exclude from this study because it was difficult to determine the level of contact. It means that participants passed by someone for a very short time, which means that they cannot be considered to have made quantitative or qualitative contact with the others. Therefore, these participants with only were excluded from this study. In order to match the number of participants for each group, each 50 participants were assigned to “contact” and “non-contact” groups for each country, totally 100 participants were selected for each country (USA: 50 for contact, 50 for non-contact; Russia: 50 for contact, 50 for non-contact; Japan: 50 for contact, 50 for non-contact, China: 50 for contact, 50 for non-contact). Among participants, 2 from USA group (n = 98, male = 57, Mean age = 23.03, SD = 2.39), 4 from Russia group (n = 96, male = 36, Mean age = 22.50, SD = 2.49), 2 from Japan (n = 98, male = 42, Mean age = 23.82, SD = 2.85), and 5 from China (n = 95, male = 23, Mean age = 22.61, SD = 2.64) were excluded from the final analysis due to being beyond two standard deviations (SD).

### Measures

#### Questionnaires

*Cultural experience questionnaire*. A cultural experience questionnaire survey was conducted to assess the quantity and quality of contact experiences with people from other countries. The questionnaire was divided into two parts: quantity of contact and quality of contact. The quantity of contact experience divided again into three sections and asked questions about contact experience status, contact path and subjective amount of contact. All participant was asked to choose between yes or no for the contact state and they were asked to choose between 5 options for the contact path. Examples of the five options are as follows, consisting of what was developed and used in the study of Koreans exploring their national image of Russia [33]: (1) acquisition of information through documented information such as books, (2) exchange through indirect methods means such as telephone, e-mail, and the internet, (3) exchange through language, (4) short-term residential experience due to travel or visiting acquaintances and exchanges through the language of the relevant country, (5) long-term residential experience due to study abroad or language training program, and exchange through the language of the relevant country. For the subjective amount of contact, a 5-point Likert scale (1 = very few, 5 = a lot) was presented to evaluate the subjective degree of individual contact experience. The quality of contact experiences was presented with a total of seven questions consisting of one question each for “pleasure”, “competitiveness”, “necessity”, “intimacy”, and three questions for “spontaneity”. Each question was measured on a 5-point Likert scale (1 = not at all, 5 = very much) and the score range was 7 to 35. The higher score means that the quality of the contact experience is positive. These items were used by previous study [34] and were empirically tested for reliability and validity.

*National Image Questionnaire*. The National Image Questionnaire which composed and used in the previous study [33] was partially modified and used according to the purpose of this study. The questionnaire consisted of four questions about the participants’ overall perception of each country and its role in security and unification issues on the Korean Peninsula and each question was repeated with a different country name. Examples of the questions are as follows: “What is your usual image of (each country)”, “What is the image of (each country) that the group belongs to you has?”, “What do you think about (each country)’ role and influence in the international community today?”, “What do you think about (each country)’ role and influence in relation to security issues on the Korean Peninsula?”. Each question was measured on a 5-point Likert scale (1 = very negative, 5 = very positive) and the score range was 4 to 20. The higher score means that the image of national is positive.

### Procedure

This study was approved by the University Institutional Review Board (NO. 1041078-201903-HRSB-094-01) and was conducted from November 2019 to January 2020. When participants arrived at the laboratory, they received information about the study procedure and completed the informed consent form. The consent form was described the problematic situations that may occur while participating in this study, and guided that participant can quit at any time without any disadvantages if they feel uncomfortable during participation. This was followed by a completion of the self-questionnaires including the Culture Experience Questionnaire, National Image Questionnaire, and demographic information questionnaire. The entire procedure took approximately 15 minutes, and the participants were debriefed and provided with monetary compensation.

### Data analysis

The data was analysed using SPSS version 25.0. National images were compared among countries after converting national image scores into Z scores (a standard practice) [35]. Z scores were positive if they contained positive values (+), but negative if they contained negative values (-). Frequency and descriptive statistics analyses were conducted to identify participant characteristics. Planned *t*-tests were conducted to examine differences in national images according to both quantity and quality of contact. The main contrasts of interest in this study were between contact/non-contact and positivity/negativity.

## Results

### Group characteristics

To confirm the group characteristics of this study, frequency analysis was conducted. Table 1 shows information on the group characteristics of the participants, except for those who were excluded from the analysis due to being beyond two standard deviations (SD). Among the participants, two from the Japanese group, two from the USA group, four from the Russian group, and five from the Chinese group were excluded from the final analysis, and finally 387 participants were analyzed. The number of respondents to the contact path by country was as follows. Contact through documented information was found to be 2 in Russia and USA respectively, and 5 in China. For the contact by indirect methods as internet was found to be 2 in China and Russia. Most of the other contact path were for official language use, short-term residence, and long-term residence. Specifically, in the case of Japan, the participants reported more experiences of short-term residence, the USA was more likely to be long-term residence, and China was the most frequently contacted using official language.

**Table 1:** Group characteristics in four countries (n = 387).

Type of variables	Subtypes	Russia	USA	Japan	China
		(n = 96)	(n = 98)	(n = 98)	(n = 95)
Gender	Male	36 (37.50%)	57 (58.16%)	42 (42.86%)	23 (24.21%)
	Female	60 (62.50%)	41 (41.84%)	56 (57.14%)	72 (75.79%)
Contact Status	Contact	46 (24.47%)	48 (25.53%)	48 (25.53%)	46 (24.47%)
	Non-contact	50 (25.13%)	50 (25.13%)	50 (25.13%)	49 (24.62%)
Contact Path	None (non-contact)	50 (25.13%)	50 (25.13%)	50 (25.13%)	49 (24.62%)
	DI	2 (22.22%)	2 (22.22%)	0 (0%)	5 (55.56%)
	IM	2 (50.00%)	0 (0%)	0 (0%)	2 (50.00%)
	OL	13 (18.12%)	14 (20.59%)	17 (25.00%)	24 (35.29%)
	STR language relevant country	15 (22.39%)	15 (22.39%)	25 (37.31%)	12 (17.91%)
	LTR language relevant country	14 (35.00%)	17 (42.50%)	6 (15.00%)	3 (7.50%)
Subjective amount of contact	Contact (Mean, SD)	2. 83 (1.08)	3.01 (1.08)	2.71 (0.94)	2.57 (1.03)
	Total (Mean, SD)	1.35 (1.60)	1.50 (1.71)	1.33 (1.51)	1.24 (1.47)
Quality of contact	Contact (Mean, SD)	28.74 (3.26)	2.88 (3.90)	24.98 (4.15)	24.63 (5.05)
	Total (Mean, SD)	13.77 (14.61)	12.18 (12.79)	12.24 (12.88)	11.93 (12.86)
National image	Mean (SD)	11.12 (2.27)	14.26 (2.63)	11.45 (2.75)	10.59 (2.50)

*Note*: DI = through documented information, IM = through indirect methods, OL = through official language, Korean or body language, STR = short term residential, LTR = long term residential

### Correlation between subjective amount of contact, quality of contact, and national image

To confirm the correlation between subjective amount of contact, quality of contact, and national image, Pearson’s r was conducted. It was found that there was a significant correlation between subjective amount of contact and quality of contact (*r* = .101, *p* = .047), and between subjective amount of contact and national image (*r* = .894, *p* < .001). On the other hand, there was no statistical significance between quality of contact and national image (*r* = .098, *p* = .053), but a weak tendency was found (Table 2).

**Table 2:** Correlation between variables.

Variables	NI	QC	SAC
NI	-		
QC	0.098	-	
SAC	0.894^***^	0.101^*^	-

*Note*: NI = national image, QC = quality of contact, SAC = subjective amount of contact

Note:

* p < .05

*** p < .001

### National image according to the quantity of direct contact experience

**Fig 2:**
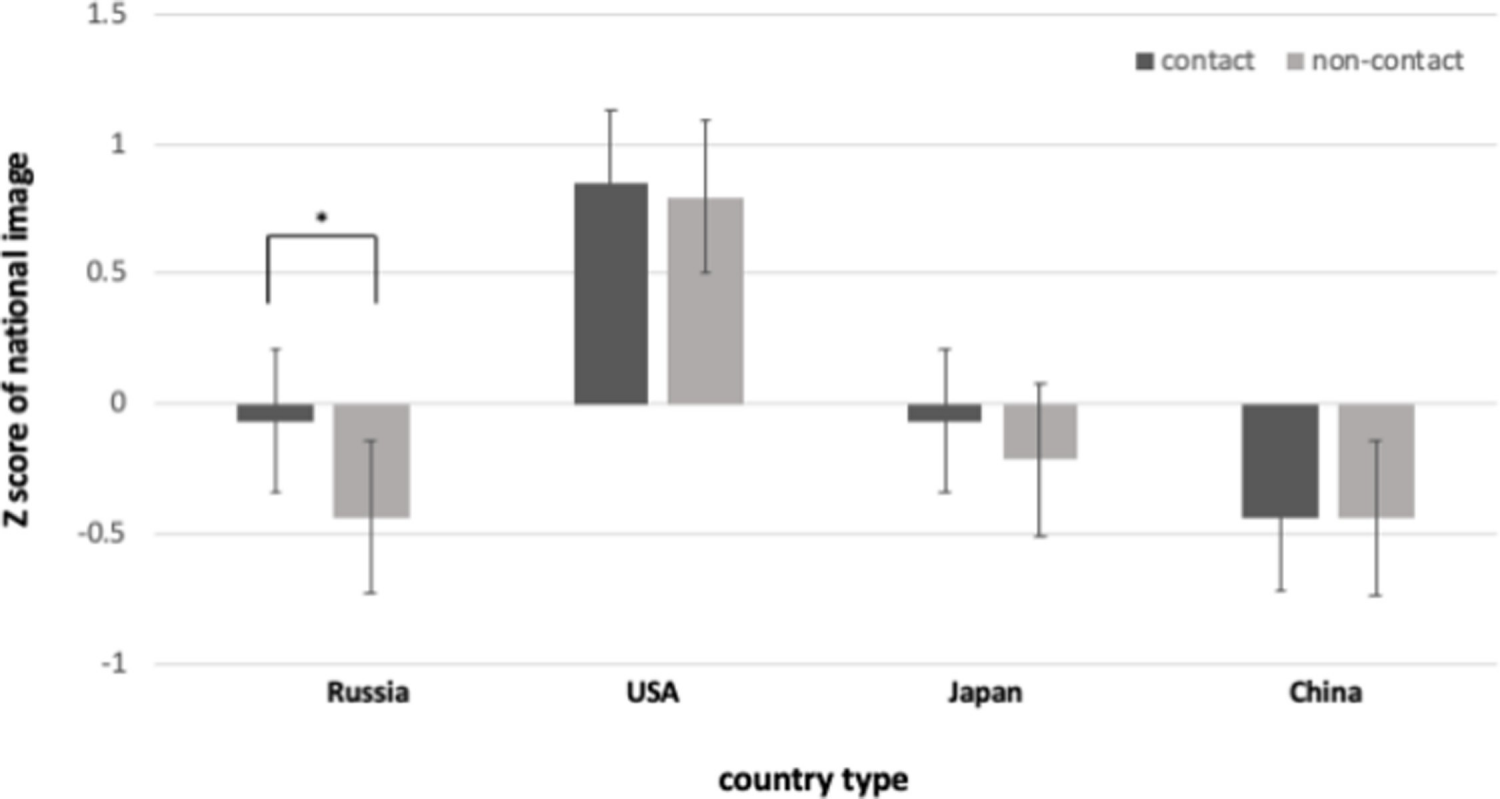
Z-scores of national images according to the quantity of contact. (*Note*: * p < .*05)*.

To confirm how the national image differs according to the quantity of contact with each country, a two-way ANOVA was conducted after the contact status and each country type were set as independent variables, and the national image as dependent variables. There was no significant interaction between the country type and contact status for national image (*F*(3, 379) = .84, *p* = .47, *ŋ*^*2*^ *=* .*005*). However, there was a significant main effect on country type (*F*(1, 379) = .40.41, *p* < .001, *ŋ*^*2*^ *=* .*24)*. To detect different national images for each country, pot-hoc comparison with Tukey was conducted. The national image was significant positive in USA compared to Russia, Japan, and China (all *p* < .001). Additionally, planned *t*-test was conducted to detect different national images for each country depending on the presence or absence of contact experience. For Russia, there was a significant difference in national image between contact and non-contact groups (*t*(94) = 2.38, *p* < .05, *Cohen’s d* = .49). In other words, the group that had contact experience with Russians held a more positive attitude towards Russia than the USA, Japan, and China (*t*(96) = .29, *p* = n.s.; *t*(96) = .77, *p* = n.s.; *t*(93) = -.01, *p* = n.s.) (Fig 2).

### National image according to the contact path experience and subjective amount of contact

A one-way ANOVA was conducted to determine differences in the images of each country across groups in terms of contact path (i.e., the quantitative aspect of the contact experience). Japan and China showed significant differences in national image in terms of contact path [*F*(2, 45) = 3.44, *p* < .05, *ŋ*^*2*^ = .13; *F*(2, 36) = 3.48, *p* < .05, *ŋ*^*2*^ = .16]. However, Russia and the USA showed no significant differences [*F*(2, 37) = 0.02, *n*.*s*.; *F*(2, 43) = 1.63, *n*.*s*.]. Tukey post-hoc analyses was conducted on Japan and China (which revealed significant differences; all results were *p* < .05). For Japan, there were significant differences between the group with experience of contact with Japanese people in Korea and the group with a short-term experience in Japan (*t*(40) = -3.028, *p* < .05). In other words, the group with short-term residencies in Japan had a more positive image of the country compared to the group that only had experience with Japanese people in Korea. For China, there were significant differences between the group with short-term residencies in China and the group with long-term residencies in China (*t*(13) = -2.712, *p* < .05). That is, people who had lived in China for long periods of time (e.g., studying abroad) held more positive national images of China than those who had been in China only for short periods of time (e.g., traveling).

To test for different images of each country in terms of the quantity of subjective contact a one-way ANOVA was conducted. Results showed no differences in national image in terms of the degree of subjective contact for Russia, Japan, China, and the US [*F*(2, 43) = 0.77, *n*.*s*.; *F*(2, 45) = 0.37, *n*.*s*.; *F*(2, 43) = 1.17, *n*.*s*.; *F*(2, 45) = 2.08, *n*.*s*.].

### National image according to the quality of direct contact experience

**Fig 3:**
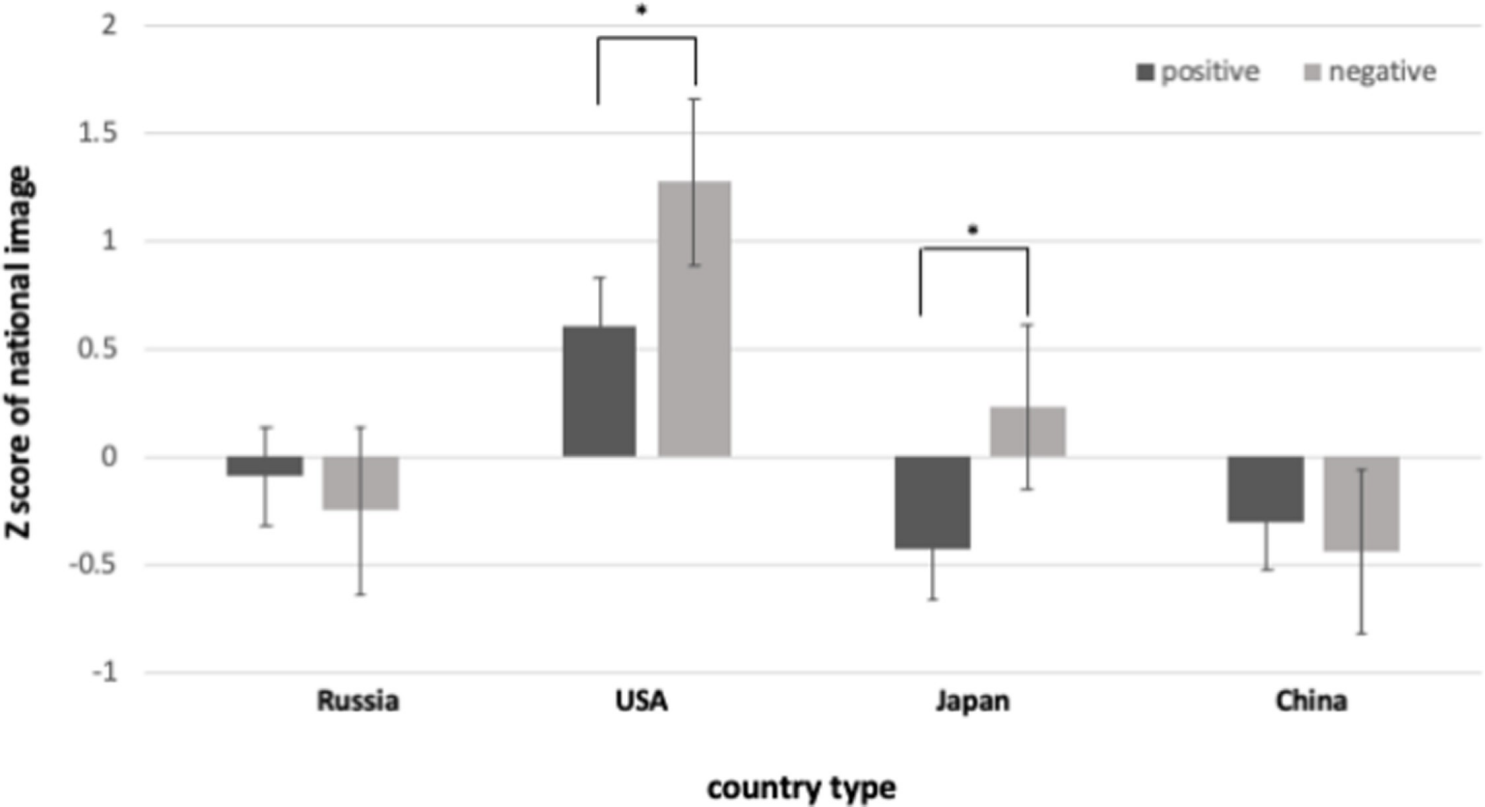
Z-scores of national images according to the quality of contact. (*Note*: * p < .*05)*.

Linear regression analysis was conducted to examine the influence of participants’ quality of contact on the national image. As a result, it was confirmed that the contact quality can significantly predict the national image (*β* = .17, *R*^*2*^ = .03, *p* = .20). In other words, when people positively evaluate qualitative experiences when they encounter other countries, the image of the country also seems to be positive. Additionally, planned *t*-tests were conducted to test for any differences in the images of each country according to the positivity/negativity of the contact experience. These elements were divided into the upper and lower 30% ranges of the contact quality expressed by the contact group. That is, the positive experience group was in the top 30%, while the negative experience group was in the bottom 30%. It was found that the group that contacted the USA and Japan had a significant difference in the country’s image depending on the type of contact quality (*t*(34) = -2.11, *p* < .05, *Cohen’s d* = -.76 ; *t*(30) = -2.08, *p* < .05, *Cohen’s d* = -.82). This means that in the case of people with the top 30% of contact quality, they have formed more positive images for each related country compared to those with the bottom 30%. On the other hand, those who contacted China and Russia showed no significant difference (*t*(29) = 0.40, *n*.*s*.; *t*(30) = .40, *n*.*s*.) (Fig 3).

## Discussion

This study examined how the degree and level of contact with countries that have maintained continuous alliances with Korea since the Korean War and countries that have maintained strategic partnerships as needed affect the image of each country. To this end, this study analyzed the relation with the national image by measuring the quantity and quality of contact of an individual with each country. The quantity of contact included an evaluation of the individual’s subjective amount of contact, contact path, and contact status, and the quality of contact was measured as an evaluation for the pleasure, competitiveness, intimacy, spontaneity, and necessity when contacting each country’s culture.

First, regardless of the type of country, the national image was highly correlated with the degree of subjective contact evaluated by individuals, but there was a weak tendency with contact quality. In other words, evaluating that an individual has subjectively had more contact with a country is more relevant to positively recognizing the image of the country concerned than evaluating the qualitative aspects of contact with one country through specific items of pleasure, competitiveness, necessity, intimacy, and spontaneity. Second, there was no significant interaction between the country type and contact status for national image, however, different national images for each country were detected. The national image of USA was significant positive compared to Russia, Japan, and China. This means that Koreans, who usually had the most active alliance with USA, tend to have a positive national image of USA, regardless of whether they had direct contact with the USA. On the other hand, in the analysis conducted to determine whether the national image differs depending on the presence or absence of contact experience, the result showed that direct contact with Russia tends to be more positive for the country than non-direct contact compared to the USA, Japan, and China.

There were inconsistent results for national images among different countries. This is consistent with previous studies, thus suggesting that the effects of contact may vary according to group [21]. Direct contact does not always have a positive effect. However, this depends on existing relations between groups. These results show that the relations between Korea and the four major nations involved in the Korean Peninsula differ according to various aspects. This may lead to inconsistent results among countries. Although South Korea has strategic alliances with both China and Russia, China tends to be closer in psychological and physical distance than Russia because it has a characteristic of sharing a geographically close and similar culture. Therefore, direct contact with Russia may have a more direct influence on changing national image because Koreans tend to lack knowledge of Russia compared to the other three countries [36]. On the other hand, traditional alliance countries (e.g., the US and Japan) tend to have friendly diplomatic relations with Korea. As a result, Koreans have a relatively positive attitude and a high likelihood of having direct contact experiences with American and Japanese citizens (e.g., through travel). Direct contact may thus be insufficient to change national images of traditional alliance countries.

As an additional example, Korea’s relations with China are close, but it is likely that it is already forming a somewhat negative national image due to its experience of conflict by similar cultures [37]. This can be difficult to change through contact experience. Affective factors are known to play a more crucial role as mediators in intergroup contact than cognitive factors [14]. That is, the strong feelings about China that have been cumulated over decades might not be overcome by the increased counter stereotypic knowledge. However, because of conducting an analysis to see if the contact path affects the difference in the national image, it was confirmed that Japan and China showed a significant difference in the contact path compared to the USA and Russia. Specifically, for Japan, the group of short-term residents in Japan tended to have a positive image of the country, and for China, long-term residents (e.g., studying abroad) showed more positive images of China than short-term residents (e.g., traveling). In other words, even if a negative image of a specific country has been formed, the possibility that the image can change positively when staying for a long time within the relevant culture is suggested.

The results of this study showed that Koreans who had positive contact with individuals from alliance countries had more positive national images of those countries than those who had negative contact. For strategic partner countries, Koreans who had contact experiences with Russians showed a more positive national image of Russia compared to those who did not. However, no such differences were detected regarding China. There were no significant differences in national image perceptions for either country based on the quality of contact. However, in the case of China, long-term residence experience may modify the national image.

This study found that the quantity and quality of contact (two factors related to direct contact experience) had different effects among countries neighbouring the Korean Peninsula. At present, international tensions are complicated by the potential denuclearisation of North Korea. In this situation, it is possible to facilitate international exchanges and relations by confirming the national images held about each country by Koreans in their 20s who are active in such dealings. The current movement for global peace is proceeding rapidly. The reduction of international tensions will alleviate various negative international relations and situations. These changes can alter the national images that Koreans hold about the four main countries active on the peninsula. It is therefore necessary to determine current national perceptions about the four countries in accordance with future changes.

### Limitations

The limitations of this study are as follows. First, we measured the quantity and quality of contact equally for each country. However, actual meanings may differ because each country had different weights for these items. For example, it may be more meaningful to meet once with a Russian who is both psychologically and physically distant and resides in an unfriendly country than meet 10 times with a Japanese. Furthermore, the cultural experience questionnaire used to measure the amount and quality of contact experience in this study was written with a design developed by the researchers themselves, and its validity was not sufficiently secured. However, since the existing questionnaire measuring the quantity and quality of contact experience was developed based on the questionnaire about cultural experience already used in the previous study [33], it is judged to be reliable to some extent. Therefore, standardised measures of the quantity and quality of contact are needed in future research. Second, it is difficult to generalise experimental results from one age group to other age groups (this study was conducted only among participants in their 20s). Since relations and situations involving other countries change over time, national images may also vary. It is thus necessary to examine the national images of various ages in future research. Third, this study was limited to the national images of the US, Japan, China, and Russia. It can also be meaningful to assess the national images of other countries neighbouring Korea. Finally, only the national images held about neighbouring countries by Koreans were confirmed. Future research should examine the national images that these countries hold about Korea.

## Supporting information

S1 Data (XLSX)
